# The impact of high-intensity interval training exercise on breast cancer survivors: a pilot study to explore fitness, cardiac regulation and biomarkers of the stress systems

**DOI:** 10.1186/s12885-020-07295-1

**Published:** 2020-08-20

**Authors:** Kellie Toohey, Kate Pumpa, Andrew McKune, Julie Cooke, Marijke Welvaert, Joseph Northey, Clare Quinlan, Stuart Semple

**Affiliations:** 1 Research Institute for Sport and Exercise, University of Canberra, Canberra, 2601 Australia; 2 Discipline of Sport and Exercise Science, Faculty of Health, University of Canberra, Canberra, 2601 Australia; 3 Health Research Institute, University of Canberra, Canberra, 2601 Australia; 4 Prehabilitation, Activity, Cancer, Exercise and Survivorship (PACES) Research Group, University of Canberra, Canberra, 2601 Australia; 5 School of Health Sciences, University of KwaZulu-Natal, Durban, 400 South Africa; 6 Statistical Consulting Unit, Australian National University, Canberra, 2600 Australia

**Keywords:** Exercise, Cancer, Immune function, Biomarkers, High-intensity, Health, Stress

## Abstract

**Background:**

Cardiovascular disease (CVD) remains the largest cause of death in breast cancer survivors. The aim of this study was to explore the impact of exercise intensity on aerobic fitness and autonomic cardiac regulation (heart rate variability (HRV)) and salivary biomarkers of the stress systems (HPA-axis, cortisol; sympathetic nervous system, α-amylase) and mucosal immunity (secretory(s)-IgA), markers of increased risk of CVD in breast cancer survivors.

**Methods:**

Participants were randomly assigned to; 1) high intensity interval training (HIIT); 2) moderate-intensity, continuous aerobic training (CMIT); or 3) a wait-list control (CON) for a 12-week (36 session) stationary cycling intervention. Cardiorespiratory fitness (VO_2peak_), resting HRV and salivary biomarkers were measured at baseline 2–4 d pre-intervention and 2–4 d post the last exercise session.

**Results:**

Seventeen participants were included in this study (62 ± 8 years, HIIT; *n* = 6, CMIT; *n* = 5, CON; n = 6). A significant improvement (*p* ≤ 0.05) was observed for VO_2peak_ in the HIIT group; 19.3% (B = 3.98, 95%CI = [1.89; 4.02]) and a non-significant increase in the CMIT group; 5.6% (B = 1.96, 95%CI = [− 0.11; 4.03]), compared with a 2.6% (B = − 0.64, 95%CI = [− 2.10; 0.82]) decrease in the CON group. Post intervention improvements in HRV markers of vagal activity (log (ln)LF/HF, LnRMSSD) and sympathetic nervous system (α-amylase waking response) occurred for individuals exhibiting outlying (> 95% CI) levels at baseline compared to general population.

**Conclusion:**

High intensity interval training improved cardiovascular fitness in breast cancer survivors and improved cardiac regulation, and sympathetic nervous system (stress) responses in some individuals. High-intensity interval training was safe and effective for breast cancer survivors to participate in with promising results as a time efficient intensity to improve physical health and stress, reducing CVD risk.

**Trial registration:**

This pilot study was retrospectively registered through the Australian New Zealand Clinical Trials Registry (ANZCTR): ACTRN12620000684921.

## Background

Cardiovascular disease (CVD) remains the largest cause of death in breast cancer survivors [1]. Exercise has been shown to reduce both physiological and psychological stress as well as CVD risk in cancer, but the specific dose and intensity of exercise required to elicit these benefits is unclear [2–4]. Breast cancer is the leading cause of death in women aged 20–50 years, with diagnosis numbers growing each year [5]. The World Health Organization (WHO) reported 2.08 million cases of breast cancer worldwide in 2019, a major contributor to the global burden of disease [6]. Women diagnosed with breast cancer often experience complications after surgery such as breast cancer related lymphoedema, axillary web syndrome, and cancer-related fatigue [7–9]. They also commonly suffer from long term treatment related side effects such as peripheral neuropathies and reduced quality of life [7, 10–13]. These side effects mean that women with breast cancer often present with low baseline fitness, strength, and quality of life (QoL) and could achieve large physiological and psychological adaptations from performing regular exercise, translating into a reduction in risk factors for CVD [14–16] and better health outcomes.

Chronic stress has been defined as a maladaptive state that is associated with altered immunity, hypothalamic pituitary adrenal (HPA) axis, and autonomic nervous system (ANS) functioning [17]. Both the independent and interactive effects of the immune system, HPA axis and ANS are key to understanding adaptive and maladaptive psychological and physiological responses to stress [18–20]. While research is still limited, HPA axis and ANS dysfunction, as well as suppression of immunity and low-grade inflammation are associated with increased CVD risk, depression and mortality in breast cancer survivors [21–23].

Protection from pathogens may be compromised in cancer patients for whom radiation, chemotherapy, surgery or effects induced by the cancer itself lead to immunosuppression [24–28]. Immunity, specifically cell-mediated immunity, is critical for defence against some types of tumours and has been shown to be decreased in metastatic breast cancer patients, related to a dysfunctional HPA axis (our central stress response system) [23]. In addition, older people are less resistant to pathogenic microorganisms, as they experience age-related decreases to immune function [29]. Research has shown that regular exercise can stimulate the immune system in older people, which increases resistance to infections [30–32]. In addition to enhanced immunity to pathogens, regular exercise also has the potential to be anti-inflammatory in nature, reflecting a mechanism via which low-grade inflammation and associated CVD risk of aging can be reduced [33, 34]. Therefore, exercise is a potential intervention to prevent a decline in immunity, reduce low-grade inflammation and CVD risk in breast cancer survivors as they age.

Autonomic cardiac regulation, as determined by the non-invasive measurement of heart rate variability (HRV) can be used as a measure of ANS activity, specifically the parasympathetic nervous system (PNS), at rest and in response to physiological and psychological stress. Decreased ANS activity, is reflected by a decrease in resting HRV or HRV reactivity to stress, and reduced ability to regulate the sympathetic nervous system (SNS). This condition of the ANS, with a decreased regulation of the SNS, is associated with CVD factors such as physical inactivity, hypertension, diabetes, and CVD. Decreased resting and reactivity HRV also occurs in response to chronic stress and is associated with high fatigue levels and reduce QoL [35, 36]. Recent research indicated that autonomic dysfunction is prevalent in cancer survivors [37]. Cancer and associated treatments negatively impact ANS activity, contributing to increased cardiovascular morbidity and mortality within the cancer population [38]. These treatments could impact the function of the ANS by damaging the nerve fibres and interfering with messages between the brain and the ANS [39–41]. This occurs by a combination of sympathetic overactivity and parasympathetic underactivity negatively impacting health by causing adverse effects such as hypertension and CVD [42, 43]. Chemotherapy could potentially impact acetylcholine levels [44] directly impacting the PNS, suggesting that the vagus nerve could be implicated via the same mechanism caused by chemotherapy. These changes may be reflected in lower resting HRV in breast cancer survivors. The underlying mechanisms for this change and the effect of exercise on mitigating negative changes seen in the breast cancer population requires further research.

Currently, the impact of exercise intensity on improving resting HRV and salivary biomarkers of stress and mucosal immunity in cancer survivors is unclear. A better understanding could help improve health outcomes by reducing stress related physical changes and psychological factors experienced by breast cancer survivor’s due diagnosis and treatment toxicity. This knowledge will assist in informing the development of individualised exercise strategies to improve health factors and reduce risk for CVD [45] in the cancer population. The current pilot randomised controlled trial was designed to explore the impact of high-intensity interval training (HIIT) on cardiovascular fitness and markers of cardiac regulation (HRV), sympathetic nervous system activity (salivary (s) α-amylase (s-AA)), HPA axis (salivary cortisol (s-cortisol)), and mucosal immunity (salivary immunoglobulin A (s-IgA)) in breast cancer survivors.

## Methods

### Study design and participants

**Fig. 1:**
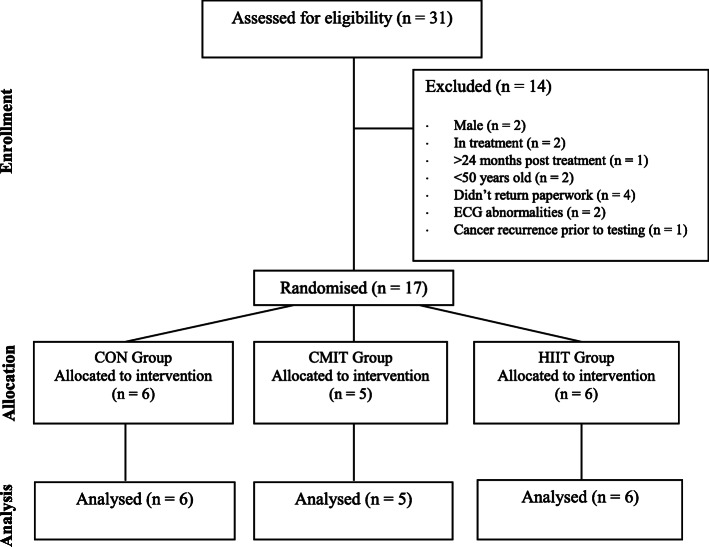
Consort diagram

This study was a pilot three-arm, 12-week randomised control trial (RCT) with pre and post measures. Participants were included in this study if they were; (1) females between the ages of 50 and 75 years, (2) sedentary as classified by the American College of Sports Medicine [46], (3) were within two years post cancer treatment and (4) did not take blood pressure medication (angiotensin-converting enzyme inhibitors or angiotensin receptor blockers or calcium channel blockers or beta blockers), (5) did not have brain or bone metastasis or (6) a diagnosis of secondary cancers and (7) were able to perform the exercise sessions on a stationary cycle ergometer (Monark 828E Ergometer) [47] (Fig. 1). The University of Canberra Human Research Ethics committee approved this study (13–153). This pilot study was retrospectively registered through the Australian New Zealand Clinical Trials Registry (ANZCTR): ACTRN12620000684921.

### Randomisation, stratification, concealment, and allocation

Following the baseline testing, participants were randomly allocated to one of three groups: high intensity interval training (HIIT); continuous moderate intensity training (CMIT); or control (CON). A concealed, computer generated sequence of numbers in blocks of variable sizes [3, 6, 9] in a 1:1:1 ratio for the three intervention groups stratified by age (< 60 years and ≥ 60 years) was generated by a researcher not involved (blinded) in the study. After baseline testing a sealed envelope with the group allocation was given to the participant. Study participants were told the overall aim of the study was to compare the effects of different physical exercise interventions on health-related outcomes.

### Intervention groups

#### Exercise groups

Participants in the two exercise interventions attended the University of Canberra laboratory three times per week for twelve weeks (up to 36 sessions). Participants could choose from a series of scheduled timeslots where supervision was provided across the week and where compliance could be recorded. Each session was conducted on the Monark cycle ergometer and lasted 20–30 min depending on the allocated intervention group.

Sessions were fully supervised by an experienced Accredited Exercise Physiologist or Accredited Exercise Scientist. Participant’s heart rate (HR) was continuously measured and recorded during all exercise sessions using a heart rate monitor (Polar FT40, Finland). Rating of perceived exertion (RPE) was monitored and recorded throughout each session (Borg 6–20) [48]. Exercise sessions started and finished with a 5-min warm up and cool down, completed on the cycle ergometers at ~ 50% of their maximal power (watts) achieved in the baseline incremental exercise test.

The CMIT group cycled for 30 min in total, with 20 min at 55–65% of their maximal power. The workload was adjusted over 12 weeks within this range to ensure their RPE remained between 9 and 13 on the Borg scale [49]. The HIIT group completed seven 30 s intervals (as hard as they could) with 2 min of active recovery between each. Participants were instructed to increase their cadence to between 95 and 115 RPM to ensure consistent performance. Participants initially completed four intervals in each session, and this was gradually increased to achieve the target of seven intervals by week four.

#### Control group

Participants in the wait listed control group (CON) were asked to continue with their current lifestyle for 12 weeks after the baseline tests. After completion, the participants from the CON group were offered the 12 week fully supervised intervention.

### Testing protocols

Participants were asked not to consume food or caffeine or participate in exercise within two hours prior to pre-and post-testing. Assessments were carried out within the 2–4 days prior to commencement of the program and within 2–4 days following completion. HRV and salivary biomarker measures were taken prior to cardiorespiratory fitness testing.

### Cardiorespiratory fitness

#### Assessment of maximal aerobic power

A maximal graded incremental cycling test was conducted to determine VO_2 Peak_, intervention relative intensity and pre and post intervention fitness levels (High-Performance Ergometer, Schoberer Rad MeBtechnik, Germany). Participants respired through an oro-nasal mask (Hans-Rudolph 7450 Series V2™ Mask, CareFusion, France), breath by breath cardiopulmonary data (Vyntus CPX, Metabolic Cart, Jaeger, Germany) were measured to calculate VO_2Peak_ in the cardiopulmonary exercise test. Throughout the test an Accredited Exercise Physiologist monitored participants with 12-Lead electrocardiogram (ECG). Blood pressure was assessed via sphygmomanometry and was recorded every two minutes.

The protocol commenced with a five minute warm up at 20 watts [50]. Thereafter, the workload was increased by ≤20 watts each minute [50] until three of the following criteria [51] were reached: 1) no change in oxygen consumption with increasing workload, 2) respiratory exchange ratio > 1.1, 3) heart rate within 10% of age predicted maximal heart rate or, 4) inability to maintain pedalling cadence. Participants self-selected peddling cadence > 60 rpm. In addition, exercise was terminated on the presentation of volitional fatigue, abnormal changes in blood pressure, or ECG abnormalities.

### Cardiac regulation and biomarker of stress

#### Heart rate variability

A Suunto watch and chest belt (Suunto model t6, Finland) was fitted to measure R-R intervals. Each belt was interfaced with the Suunto t6 watch for purposes of monitoring continuous R–R intervals [52]. Each participant sat quietly on a chair in an upright position for 10 min prior to the commencement of HRV recording. Although HRV is higher seated than supine, the seated posture was selected for its practicality and convenience [53]. R–R interval recording lasted 5 min and these were then transferred to Kubios HRV analysis software (Kubios heart rate variability software version 2.0; Biosignal Analysis and Medical Imaging Group, Department of Physics, University of Kuopio, Kuopio, Finland) for the analyses of time and frequency HRV domains. Participants’ respiratory rate during the recordings was not controlled for as there is a lack of consensus on the influence of controlled versus non-controlled breathing on HRV parameters, particularly at rates < 10 breaths/minute [54]. The protocol was carried out in accordance with the Task Force of the European Society of Cardiology and the North American Society of Pacing and Electrophysiology standards for measurement of short-term HRV [55]. One of the recordings in the CON group could not be analysed due to > 20% R-R interval artefacts over the duration of the recording [52].

#### Saliva collection and analysis

Saliva samples (s-AA, s-IgA and s-cortisol) were obtained using IPRO Oral Fluid Collection (OFC) kits that were labelled and provided to each participant. The OFC kits collect 0.5 mL of oral fluid and contain a colour changing volume adequacy indicator within the swab, giving collection times typically in the range of 20–50 s [56].

Baseline saliva samples were collected at two-time points on the same day at home, two days before and after the intervention commenced and ended (immediately upon waking whilst still in bed and 30 min post waking) [57]. The participants received training on the saliva collection procedure during their first visit to the laboratory. They were requested to adhere as closely as possible to the standardised collection guidelines, which was carried out in their home [57, 58]. Participants recorded the time each saliva sample was collected. All samples were frozen immediately after collection in home freezers and kept frozen until reaching the laboratory, upon which they were stored at − 20 °C until analysis.

### Statistical analysis

The data were analysed with a general linear mixed model using the R package lme4 (R Core Team 2018). A random intercept for participants was included to account for intraindividual dependencies and interindividual heterogeneity. This also allowed for individual baseline adjustment. All models were estimated using Restricted Maximum Likelihood. Visual inspection of residual plots did not reveal any obvious deviations from homoscedasticity or normality. *P*-values were obtained using Type II Wald F tests with Kenward-Roger degrees of freedom as implemented in the R package car [59]. Statistical significance was determined on *p* ≤ 0.05, in addition confidence intervals (CI) were assessed whether they included zero or not. Results are reported as mean estimates and 95% confidence intervals. The natural log was initially calculated and analysed for HRV parameters before the above statistical analyses were carried out. A biofeedback manual cleanup process was carried out for the HRV data using the Kubios protocol [60].

## Results

### Participants and adherence

All participants who were randomised completed the study (*n* = 17). Thirty-one participants applied to be part of the study and 14 were either not eligible (*n* = 10) or failed to respond (*n* = 4). Participants completed baseline testing before being randomised into the HIIT (*n* = 6), CMIT (*n* = 5) or CON (n = 6) (Fig. 1). Participants diagnosed with breast cancer within the prior 24 months. The mean age of participants was 62 ± 8 years, with a BMI of 26.30 ± 4.39 kg/m^2^ (Table 1). Participants were similar at baseline for age and treatment types (*p* > 0.05). Baseline values were similar for all variables across the three groups except for s-IgA, which was lower in the HIIT group compared to the CON group (B = -308.23, 95%CI = [− 555.06; − 61.41]). CMIT was significantly higher at baseline for; log very low frequency (LnVLF) (F (2, 12) = 5.23, *p* = 0.02, B = 1.95, 95%CI = [0.11; 3.79]) and non-significant for log high frequency (LnHF) (F (2, 12)=1.21, *p* = 0.07, B = 2.33, 95%CI = [0.04; 4.62]) compared to the CON group. Adherence was similar between the exercise groups (HIIT and CMIT) (percentage of sessions attended: 78.7 ± 13.2% vs 79.4 ± 12.0%; *p* = 0.93).

**Table 1:** Participant Characteristics

	**Control (*n*** **= 6)**	**CMIT (*n*** **= 5)**	**HIIT (*n*** **= 6)**
Age (y)	61 ± 7.92	65 ± 7.68	60 ± 8.12
Height (cm)	163.5 ± 5.20	165.6 ± 5.59	165.6 ± 5.78
Weight (kg)	75.63 ± 7.71	68.80 ± 11.48	69.48 ± 16.07
BMI (kg/m^2^)	28.5 ± 4.53	24.95 ± 2.48	25.23 ± 5.22
Body Fat (%)	45.52 ± 9.32	37.76 ± 5.61	33.60 ± 10.03
VO_2_ (ml/min^− 1^/kg^− 1^)	20.90 ± 3.10	20.74 ± 3.71	19.52 ± 3.89
**Treatment**			
Surgery	0	1 (20%)	0
Radiation	0	1 (20%)	0
Surgery + chemotherapy	1 (17%)	0	0
Surgery + radiation	2 (33%)	3 (60%)	3 (50%)
Surgery + chemotherapy + radiation	3 (50%)	0	3 (50%)

### Exercise intervention

The HIIT group’s average HR during the sessions was 150 ± 9 beats per minute (bpm) during the intervals, while the RPE was 12 ± 4. The average HR and RPE at the end of the two-minute recovery was 125 ± 12 bpm and 9 ± 6 bpm. The average HR during the sessions for the CMIT group was 136 ± 16 and RPE was 13 ± 10. Overall mean session compliance was 79% (78.7 ± 13.2% (HIIT) vs 79.4 ± 12.0% (CMIT); p = 0.93). There were no adverse events from the exercise intervention in this study. The HIIT group had a significantly higher relative HR (93.5 ± 7.1% vs. 83.9 ± 1.9%; *p* = 0.04) and non-significantly higher RPE (13.6 ± 1.8 vs.12.3 ± 1.6; *p* = 0.09) when compared to the CMIT group at the end of the last exercise session after the 12 weeks of training.

### Cardiovascular fitness

A significant difference (F_2,12_ = 6.53, *p* = 0.01) was seen in VO_2 Peak_ from pre to post intervention for the HIIT group. A 19.3% (B = 3.98, 95%CI = [1.88; 6.02]) increase for HIIT and a 5.6% (B = 1.96, 95%CI = [− 0.11; 4.03]) increase for the CMIT group, was observed compared to a − 2.6% (B = − 0.64, 95%CI = [− 2.10; − 0.82]) decrease in the CON group.

### Heart rate variability

#### Heart rate variability

**Fig. 2:**
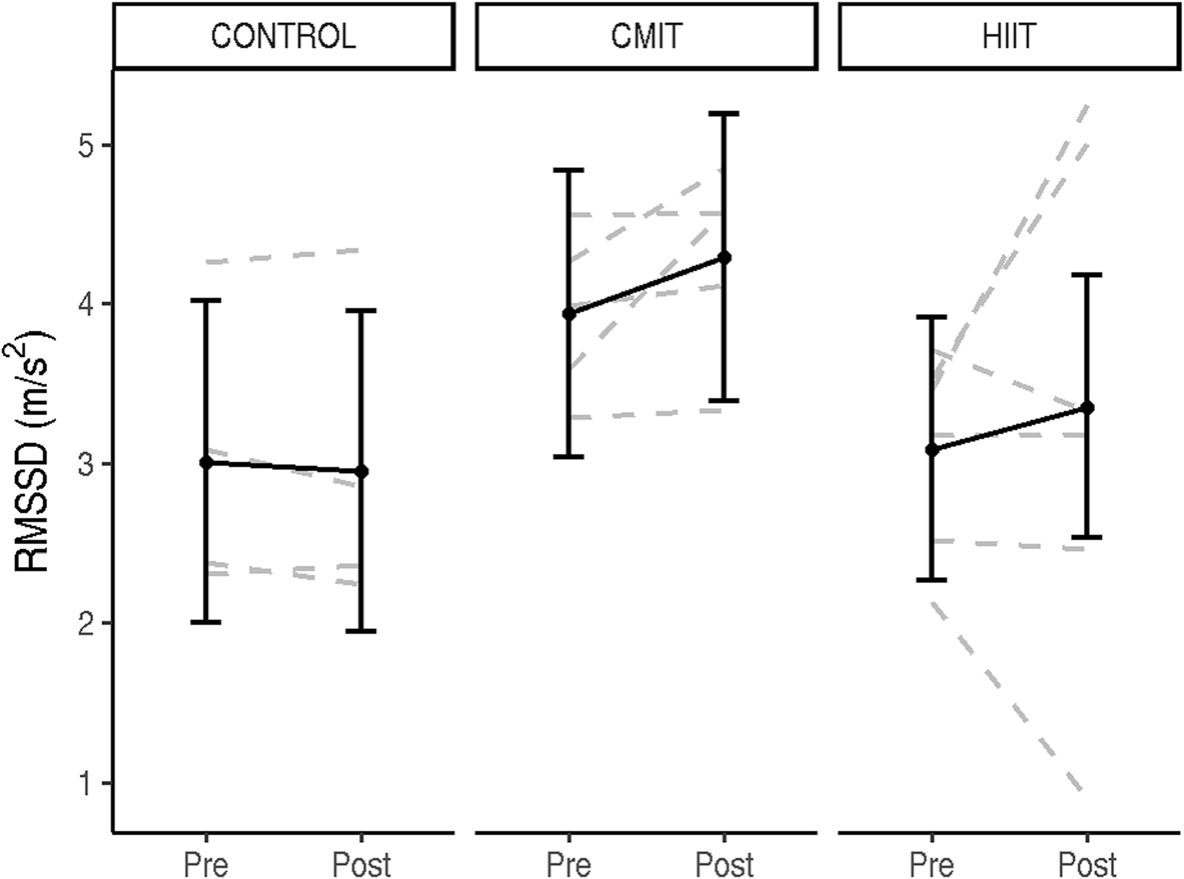
Individual responses from pre to post intervention for each group (CON, CMIT, HIIT) for heart rate variability time domains LnRMSSD in m/s^2^. Estimated group means, and 95% confidence intervals are shown in grey

Pre and post changes for HRV measures in all three groups are shown in Table 2. Individual changes for LnRMSSD are shown in Fig. 2. There were no significant changes in HRV measures from pre to post for any of the groups (all *p* > 0.05). LnVLF was significantly higher for the CMIT group compared to the other groups, both pre and post intervention (B = 1.95, 95%CI = [0.11; 3.79]).

**Table 2:** Heart rate variability changes from pre to post exercise intervention

	CON (*n* = 6)		CMIT (*n* = 5)		HIIT (*n* = 6)	
	Pre	Post	Pre	Post	Pre	Post
LnVLF	2.98 ± 0.69	2.37 ± 1.03	4.93 ± 1.11	5.06 ± 1.00*	3.78 ± 0.90	3.86 ± 2.74
LnLF	4.88 ± 0.92	3.93 ± 1.08	6.75 ± 0.73	6.85 ± 0.67	4.72 ± 2.14	5.19 ± 2.85
LnHF	4.38 ± 0.82	3.83 ± 0.75	6.71 ± 1.26	7.07 ± 0.78	4.48 ± 2.07	5.50 ± 3.13
LnLF/HF	0.51 ± 0.74	0.10 ± 0.59	0.04 ± 1.23	− 0.22 ± 0.89	0.24 ± 1.43	− 0.31 ± 0.55
Mean RR (m/s)	845.70 ± 76.01	815.66 ± 58.21	902.69 ± 37.36	792.69 ± 90.86	834.07 ± 147.40	848.11 ± 165.45
Mean HR* (b/min)	71.37 ± 6.28	73.83 ± 5.07	66.61 ± 2.84	76.47 ± 8.47	74.12 ± 14.88	73.24 ± 15.64
LnRMSSD	3.01 ± 0.90	2.95 ± 0.96	3.94 ± 0.51	4.29 ± 0.60	3.09 ± 0.63	3.35 ± 1.62

* time effect, Log (Ln), *LnVLF* very low frequency, *LnLF* low frequency, *LnHF* high frequency, *LnLF/HF* low frequency/high frequency, *RR* measure between the R waves, *LnRMSSD* root mean square of successive difference of R-R interval

### Salivary biomarkers

**Fig. 3:**
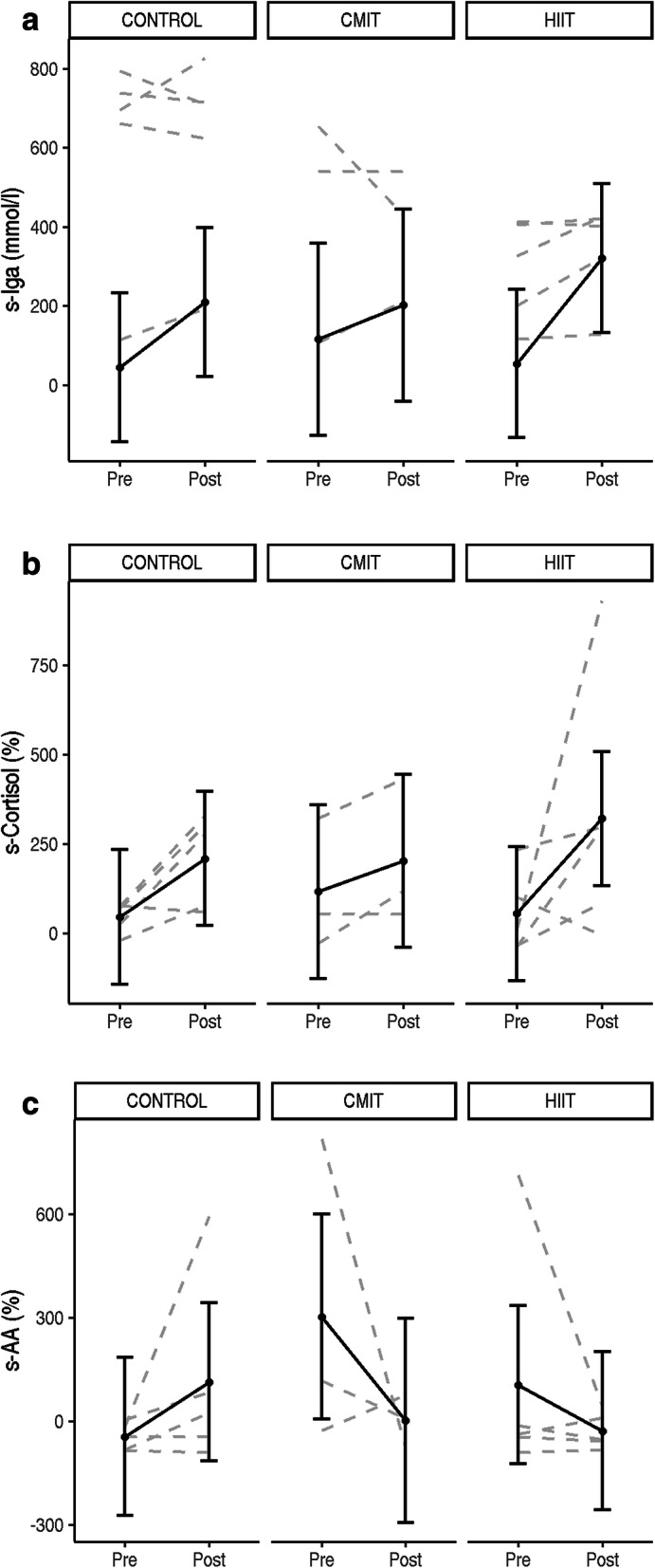
**a** 30 min post waking s-IgA individual responses from pre to post intervention for each group (CON, CMIT, HIIT). Estimated group means, and 95% confidence intervals are shown in grey. **b** Waking to 30 min post-waking (percent change) s-cortisol individual responses from pre to post intervention for each group (CON, CMIT, HIIT). Estimated group means, and 95% confidence intervals are shown in grey. **c** Waking to 30 min post-waking (percent change) s-AA individual responses from pre to post intervention for each group (CON, CMIT, HIIT). Estimated group means, and 95% confidence intervals are shown in grey

For s-IgA (30 min post waking) there were no significant differences over time or between groups (*p* > 0.1, see Fig. 3 a a for individual responses).

Overall, there was a slight increase from pre to post intervention (B = 163.65, 95%CI = [− 56.70; 384.28], *p* = 0.03) in s-cortisol expressed as percent change from waking to 30 min post waking (Fig. 3b). There were two participants within the exercise groups who demonstrated an improvement in their s-AA waking response from baseline to post intervention (Fig. 3c). However, no statistically significant group changes were observed for s-AA (*p* > 0.2).

## Discussion

The present study investigated the effect of exercise intensity on cardiovascular fitness and was the first study to measure this in combination with cardiac regulation (HRV) and salivary biomarkers of stress including mucosal immunity in breast cancer survivors. High intensity interval training improved cardiovascular fitness compared to CMIT providing preliminary support for this short and intense dose of exercise to improve health outcomes in breast cancer survivors. Non-significant improvements in cardiac vagal activity, and sympathetic nervous system responses in individuals with outlying baseline values (compared to healthy population) were detected in response to the exercise intervention, potentially reducing risk of common diseases in the cancer population such as CVD. These changes should be further investigated in longer and larger scale RCT’s.

There are limited studies reporting changes in s-AA in breast cancer, however, it has been proposed that breast cancer survivors display elevated patterns of alpha-amylase in both diurnal and acute profiles compared to healthy women [61, 62]. Only one study to date has reported changes in s-AA as a marker of stress across the chemotherapy treatment regime in two groups of breast cancer patients [63]. This study found an increase in patient stress levels as they progressed through the chemotherapy treatment cycle, and in addition in-patient stress was higher than out-patient stress [63]. Typically, s-AA would decrease significantly in the 30 min after waking, indicating a healthy response [64]. In the current study two individuals (one in the HIIT group and one in the CMIT group, Fig. 3c), did not exhibit normal s-AA waking responses. Both started the intervention with outlying, abnormal s-AA waking responses (> 95% CI) where their baseline s-AA increased by > 600% 30 min post-waking. Post-intervention these two individuals exhibited improved (normal response is ~ 50% decrease in s-AA 30 min post waking) s-AA waking responses. This indicates a positive change after an exercise intervention which should be further explored. The current study observed one individual in the HIIT group post intervention with an increase in s-AA at 30 min post waking. This result could suggest further disease or that the intervention was not long enough to exhibit a response for this individual, or more likely an error in the self-administered saliva collection test [64].

The expected cortisol diurnal rhythm is an initial increase in the first 30–60 min post waking, followed by further increases in the morning, before progressively declining into the evening. HPA dysregulation indicated by abnormal, flatter diurnal cortisol patterns (cortisol levels which do not rise during the morning or decrease in the evening) is associated with the incidence and progression of breast cancer [65, 66]. In some breast cancer survivors, blunted waking or diurnal cortisol response, across the day have been reported [67]. Importantly, in the current study a slight increase (non-significant) in s-cortisol (percent change) was seen 30 mins post waking in the exercise groups, this may signify improved HPA axis activity post intervention. This mechanism has significant clinical value because it represents a reduction in stress levels with regular exercise [68] in the cancer population and should be investigated further. Objective physiological stress markers are not commonly measured or taken into consideration in clinical practice to assess patients or prescribe individual exercise but could be considered as it is an early marker of the progression of future disease.

Salivary immunoglobulin A is an antigen specific antibody that mediates primary immune system responses and has a protective role against bacterial, viral and protozoal infections of the mucosa [69]. Disruptions to the immune system are highly correlated with cancer, obesity and CVD [70] but there are a lack of studies exploring mucosal immune function in breast cancer patients and survivors. Also the interaction of a diagnosis of cancer causes significant stress contributing to the reduction in immune function [71], increasing the risk for further disease. It has been advised that high intensity overtraining reduces s-IgA levels, weakening the immune response [72], instigating a risk with participating in HIIT, however, in the current study mucosal immunity was maintained in the exercise groups and sIgA did not increase.

Autonomic nervous system dysfunction, typically represented as low HRV, is prevalent in the cancer population (young adults with cancer and breast cancer) [38, 73], potentially contributing to treatment related side effects, such as cardiovascular decline, inflammation, increased fatigue and decreased QoL, [38, 74] and increased risk of CVD [75]. For HRV, the time domain, root mean square of successive difference of R-R intervals (RMSSD), and frequency domain, HF band, represents cardiac vagal activity [54, 76], with higher levels reflecting higher HRV and enhanced ANS activity. The LF (low frequency) band is associated with baroreflex activity and the bilateral effect of sympathetic and vagal activity on the sinus node impacting on levels of stress experienced. It has been reported that cancer survivors, and in particular those who were older, expressed significantly lower LnRSMMD levels when compared to healthy individuals [74]. In the current study, baseline LnRMSSD was slightly below the reported healthy levels [77], and rose to healthy norms [74] in the exercise groups post intervention (Fig. 2). The changes observed in this study suggest that exercise improved ANS function, specifically vagal activity, potentially decreasing treatment related side effects and the risk of CVD.

A limitation of the current study was that participants who did not undergo chemotherapy were randomly allocated into the CMIT group. This may explain the differences observed in baseline HRV variables (in the chemotherapy and non-chemotherapy groups), considering treatment regimes when stratifying participants in future studies would be advantageous. Comparable to the current study and studies prior on cancer survivors, chemotherapy could potentially be involved in the development of abnormalities in the ANS [78] although in the current study there were no participants currently undergoing active treatment. Due to the low numbers, caution must be taken regarding the generalisability of the findings to all cancer survivors. A further limitation was that saliva collection was not observed relying on participants to remember the protocol and self-report timings. Despite these limitations, clinically important results were noted which have practical application and further clinical trials would be useful to confirm the results.

## Conclusion

This study demonstrated that HIIT improved cardiovascular fitness (compared to CMIT) in breast cancer survivors and also improved cardiac vagal activity, and sympathetic nervous system responses in individuals with outlying baseline values, potentially reducing risk of diseases such as CVD. Those participants within the normal ranges at baseline (HPA-axis, ANS and mucosal immunity) remained that way and were not negatively impacted by exercise at higher intensities. High-intensity interval training was safe and effective for breast cancer survivors to participate in with promising results as improved health outcomes were observed. Future exercise guidelines for cancer survivors should consider the use of HIIT to improve levels of fitness.

## Data Availability

The datasets used and/or analysed during the current study are available from the corresponding author on reasonable request.

## Abbreviations

HRV: Heart rate variability
HPA: Hypothalamic-pituitary-adrenal
IgA: Immunoglobulin
HIIT: High intensity interval training
CMIT: Continuous moderate intensity training
CON: Control
VO_2peak_: Peak oxygen uptake
Ln: Log
VLF: Very low frequency
LF: Low frequency
HF: High frequency
RR: Measure between the R waves
RMSSD: Root mean square of successive difference of R-R intervals
CVD: Cardiovascular disease
QoL: Quality of life
ANS: Autonomic nervous system
PNS: Parasympathetic nervous system
SNS: Sympathetic nervous system
RCT: Randomised controlled trial
